# Human cytomegalovirus suppresses Fas expression and function

**DOI:** 10.1099/vir.0.058313-0

**Published:** 2014-04

**Authors:** Sepehr Seirafian, Virginie Prod’homme, Daniel Sugrue, James Davies, Ceri Fielding, Peter Tomasec, Gavin W. G. Wilkinson

**Affiliations:** Institute of Infection & Immunity, Cardiff University School of Medicine, Heath Park, Cardiff CF14 4XN, UK

## Abstract

Human cytomegalovirus (HCMV) is known to evade extrinsic pro-apoptotic pathways not only by downregulating cell surface expression of the death receptors TNFR1, TRAIL receptor 1 (TNFRSF10A) and TRAIL receptor 2 (TNFRSF10B), but also by impeding downstream signalling events. Fas (CD95/APO-1/TNFRSF6) also plays a prominent role in apoptotic clearance of virus-infected cells, so its fate in HCMV-infected cells needs to be addressed. Here, we show that cell surface expression of Fas was suppressed in HCMV-infected fibroblasts from 24 h onwards through the late phase of productive infection, and was dependent on *de novo* virus-encoded gene expression but not virus DNA replication. Significant levels of the fully glycosylated (endoglycosidase-H-resistant) Fas were retained within HCMV-infected cells throughout the infection within intracellular membranous structures. HCMV infection provided cells with a high level of protection against Fas-mediated apoptosis. Downregulation of Fas was observed with HCMV strains AD169, FIX, Merlin and TB40.

Human cytomegalovirus (HCMV), the prototype member of the subfamily *Betaherpesviridae*, is ubiquitous in human populations worldwide. HCMV establishes a lifelong persistent infection that is normally controlled by continuous host immune surveillance. Although the vast majority of infections in the immunocompetent host appear to be benign, HCMV is a major cause of severe morbidity and mortality following congenital transmission, and in immunocompromised individuals. Studies using murine and rhesus cytomegaloviruses have shown that efficient infection, superinfection and long-term persistence *in vivo* are dependent on effective viral immune-evasion functions (Babić *et al.*, 2011; Früh *et al.*, 2013; Vidal *et al.*, 2013). HCMV also possesses an impressive array of immunomodulatory functions that are instrumental in avoiding T cells, natural killer (NK) cells, the interferon response and apoptosis.

HCMV UL36 (vICA) and MCMV m36 are positional homologues (no overt amino acid sequence homology) that suppress death receptor (DR)-mediated apoptosis by inhibiting caspase-8 activation and promoting virulence *in vivo*, respectively (Ebermann *et al.*, 2012; Skaletskaya *et al.*, 2001). In HCMV, cellular DRs are also targeted directly during infection. The laboratory strain AD169 downregulates TNFR1 from the cell surface (Baillie *et al.*, 2003) more efficiently than low-passage strains (Montag *et al.*, 2006). This inconsistency was explained when UL138 was found to stimulate surface expression of TNFR1; strain AD169 has suffered a deletion of the 15 kb region UL/*b'*, which encompasses UL138 (Le *et al.*, 2011; Montag *et al.*, 2011). HCMV thus appears to encode functions capable of acting post-transcriptionally to suppress and ‘potentiate’ TNFR1 expression. We recently demonstrated that HCMV also regulates expression of a second DR: TNF-related apoptosis-inducing ligand (TRAIL) receptor (Smith *et al.*, 2013). Although HCMV infection stimulates expression of TRAIL receptor 2 (TR2) in fibroblasts, gpUL141 binds TR2 directly to sequester the DR in the endoplasmic reticulum, thereby protecting HCMV-infected cells against both soluble TRAIL and TRAIL-dependent NK cell-mediated killing (Nemčovičová *et al.*, 2013; Smith *et al.*, 2013).

Fas is another member of the tumour necrosis factor receptor superfamily (TNFRSF), recognized as playing a major role in controlling viral infections (Itoh *et al.*, 1991; Trauth *et al.*, 1989; Yonehara *et al.*, 1989). While Fas is expressed on most cell types, its cognate ligand (FasL) is restricted to activated T, NK and dendritic cells (Nagata, 1999; Nagata & Golstein, 1995). The upregulation of FasL and TRAIL on HCMV-infected dendritic cells promotes direct killing of activated T lymphocytes, an action that may preferentially delete HCMV-specific T cells (Raftery *et al.*, 2001). Moreover, the activation of FasL on HCMV-infected retinal pigment epithelial cells may subvert neutrophil function in HCMV retinitis (Chiou *et al.*, 2001; Cinatl *et al.*, 2000). Although HCMV may exploit FasL to dampen immune responses, FasL has the potential to kill HCMV-infected cells. FasL acts by inducing a conformational change in Fas, leading to recruitment of FADD and procaspase-8, and assembly of the death-inducing signalling complex (DISC) (Kischkel *et al.*, 1995; Scott *et al.*, 2009). Caspase-8 released from DISC induces cleavage of downstream substrates including effector caspases 3 and 7, resulting in proteolysis of critical cellular components and culminating in apoptosis (Barnhart *et al.*, 2003; Salvesen & Dixit, 1997). While UL36 inhibits caspase-8, the fate of Fas during HCMV infection is unclear (Chaudhuri *et al.*, 1999).

Human foetal foreskin fibroblasts (HFFF-hTERTs) (McSharry *et al.*, 2001) were therefore infected with HCMV strain Merlin and cell surface expression of Fas tracked over the course of infection. Fas was unaffected by HCMV infection until 24 h p.i.; the cell surface downregulation detected at this time point persisted through the late phase of infection (48 and 72 h) (Fig. 1a). Consistent with Fas downregulation occurring with early kinetics, cells treated with the viral DNA replication inhibitor ganciclovir showed comparable levels of Fas downregulation, demonstrating that viral DNA replication is not required for this function (72 h p.i., Fig. 1b). Latent carriage of HCMV has been shown to protect CD34^+^ progenitor cells from FasL-mediated apoptosis through increased cIL10 secretion (Poole *et al.*, 2011). However, transfer of supernatants from Merlin-infected cells did not result in substantial downregulation of Fas at the cell surface, indicating that this function is not carried out by a soluble factor (Fig. 1c). In addition, virus inactivated by γ-irradiation did not modulate Fas expression, thus suggesting the function is attributable to a *de novo* expressed virus-encoded function rather than input virions (Fig. 1c). MHC class-I was included as an infection control; downregulation of classical MHC class-I expression is achieved by four HCMV genes (US2, US3, US6, US11) that are expressed with immediate early and early kinetics (Ahn *et al.*, 1996; Hengel *et al.*, 1996; Hesse *et al.*, 2013; Lehner & Cresswell, 1996; Park *et al.*, 2002). Downregulation of Fas and MHC class-I exhibit similar kinetics (Fig. 1).

**Fig. 1.**
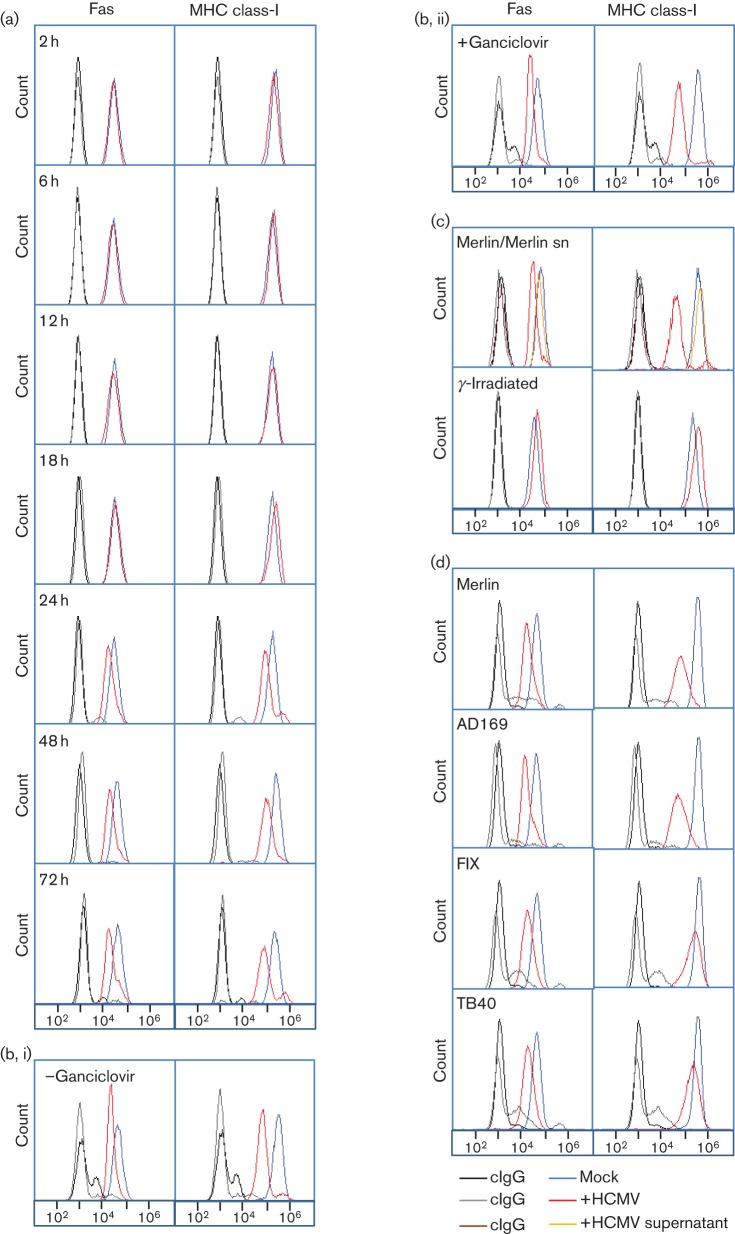
Modulation of Fas cell surface expression in cells infected with HCMV. (a) HFFF-hTERTs were infected with HCMV strain Merlin (m.o.i. 10) or mock-infected, and analysed at indicated time points by flow cytometry for cell surface Fas expression [mAb142 (R&D Systems), *n*≥3). (b) HFFFs were infected with HCMV strain Merlin (m.o.i. 10, 72 h) in the presence (i) or absence (ii) of 100 µM ganciclovir and analysed by flow cytometry for cell surface Fas expression (*n* = 3). (c) HFFF-hTERTs were incubated with supernatants (sn) of strain Merlin-infected cells (m.o.i. 10, 72 h p.i.) from which virions had been removed using a 0.1 µm filter, or were infected with HCMV strain Merlin (m.o.i. 10, 72 h) or an equivalent γ-irradiated preparation (2500 Gy) and analysed by flow cytometry for cell surface Fas expression (*n* = 3). (d) HFFF-hTERTs were infected with HCMV strains Merlin, AD169, FIX or TB40 (m.o.i. 10, 72 h) and analysed by flow cytometry for cell surface Fas expression (*n*≥3). Control IgG staining is denoted by black and grey lines for mock-infected and HCMV-infected cells, respectively.

Since HCMV exhibits an exceptionally high level of inter-strain sequence variation (Dolan *et al.*, 2004), we were interested in determining whether Fas regulation is a conserved function. The level of Fas downregulation was similar in cells infected with HCMV strains Merlin, AD169, FIX and TB40 (Fig. 1d). Comparable results were also obtained using HFFF cells and primary dermal fibroblasts (data not shown). Variation in the efficiency of MHC class-I downregulation is attributable to the fact that strains FIX (ΔUS2, ΔUS3 and ΔUS6) and TB40 (ΔUS3 and ΔUS6) are derived from BAC clones, and were deleted in the US segment to facilitate genome manipulation (Murphy *et al.*, 2003; Sinzger *et al.*, 2008).

The sensitivity of HCMV-infected cells to Fas-mediated apoptosis was ascertained by measuring the activation of effector caspases 3 and 7. Cells were infected with HCMV strains Merlin or AD169 or mock-infected and treated with FasL or a cross-linking Fas mAb, soluble TR2 or an IgM control antibody. Caspase 3/7 activity was then measured at 16 and 72 h p.i. by its capacity to cleave a luminogenic substrate in the presence of a recombinant luciferase (Fig. 2). At 16 h p.i., prior to Fas downregulation at the cell surface, there was no significant difference in caspase 3/7 activity between mock-infected and HCMV-infected cells in any of the treatment groups (Fig. 2a). However, at 72 h p.i., cells infected with strains Merlin or AD169 became less sensitive to Fas signalling induced by either FasL or Fas mAb (Fig. 2b). In addition, there was no significant difference in the level of protection imparted to cells by strains Merlin and AD169. This is interesting, since the AD169 variant that was used in this experiment carries a single amino acid substitution in the UL36 gene that abolishes the anti-apoptotic function of vICA (Skaletskaya *et al.*, 2001). HCMV infection therefore renders cells less sensitive to Fas-mediated apoptosis. This function correlates with Fas downregulation from the surface of infected cells, and can occur independently of vICA function.

**Fig. 2.**
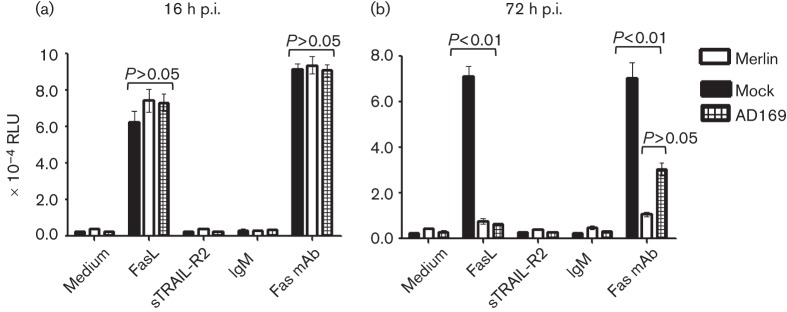
HCMV infection renders cells less sensitive to Fas-mediated apoptosis. HFFFs were infected with strain Merlin or AD169 (m.o.i. 10), or mock-infected. At 4 (a) or 60 (b) h p.i., cells were treated with cycloheximide (Sigma) at 10 µg ml^−1^ concentration and FasL (IBA-Lifesciences), Fas mAb (Beckman-Coulter), sTRAIL-R2 (control for Fas ligand) or IgM isotype control at 500 ng ml^−1^. Apoptosis was then measured at the indicated time points as caspase 3/7 activation using the Caspase-Glo 3/7 kit (Promega,). Results are presented as mean relative light units (RLU)±se (*n* = 4). *P*-values were calculated using a one-way ANOVA test and a Bonferroni post test.

Fas mRNA levels, as assessed by quantitative reverse transcriptase PCR (qRT-PCR), were not significantly affected by HCMV infection at 24, 48 or 72 h p.i. (Fig. 3a). Nevertheless, levels of Fas in total cell lysates appeared moderately reduced following infection with HCMV strains Merlin, AD169, Fix or TB40 (Fig. 3b). HCMV is known to suppress the cell surface expression of specific proteins (e.g. CD112, CD155, MHC-I, MICB, TR2, ULBP2), often by sequestering them within the cell (Cosman *et al.*, 2001; Jones *et al.*, 1996; Nemčovičová *et al.*, 2013; Prod’homme *et al.*, 2010; Smith *et al.*, 2013; Tomasec *et al.*, 2005). *N*-linked glycoproteins acquire resistance to endoglycosidase-H (EndoH) during maturation in the Golgi apparatus. Fas was clearly heavily glycosylated, as evidenced by its sensitivity to peptide *N*-glycosidase-F (PNGaseF), and was resistant to EndoH treatment±HCMV infection (Fig. 3c). Consequently, HCMV does not appear to retain newly synthesized Fas in pre-Golgi compartments. Immunofluorescence showed Fas to illuminate the surface of uninfected fibroblasts, in addition to a diffuse cytoplasmic staining pattern (Fig. 3d). In cells infected with HCMV, Fas appeared largely excluded from the plasma membrane; rather, the protein localized to extended membranous perinuclear structures (Fig. 3d).

**Fig. 3.**
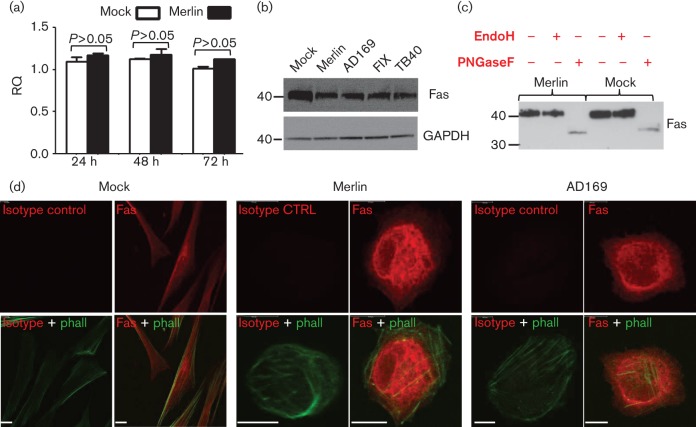
Analysis of Fas expression in HCMV-infected cells. (a) HFFF cells were infected with strain Merlin (m.o.i. 10) or mock-infected, and Fas mRNA levels were analysed at indicated times by qRT-PCR: total cell RNA was extracted (Qiagen) followed by RT-PCR using random hexamer primers (Amersham Biosciences). Resulting cDNA was analysed by qPCR using primers specific to Fas or GAPDH and SYBR green dye (applied Biosciences). Relative quantity (RQ) values were calculated by the comparative *C*_T_ method. Fas RQ values are shown normalized to GAPDH and relative to the ‘24 h p.i’. mock sample (±se, *n* = 3). *P*-values were calculated using a one-way ANOVA test and a Bonferroni post test. (b) HFFF-hTERT cells were infected with strains Merlin, AD169, FIX or TB40 (m.o.i. 10, 72 h), or mock-infected. Total cell lysates were analysed by Western blot (ERP5700, Abcam, *n*≥3). (c) Cell lysates equivalent to (b) were treated with EndoH (New England Biolabs) or PNGaseF (New England Biolabs) and analysed by Western blot (*n*≥3). (d) HFFF-hTERT cells were infected with strains Merlin or AD169 (m.o.i. 10, 72 h), or mock-infected, and Fas expression was visualized by immunofluorescence [mAb142 (R&D Systems), *n*≥3, shown in red in top panels]. In the lower panels, outlines of cells were visualized with phalloidin-AF488 (phall; Invitrogen) and overlaid with Fas staining. Scale bars, 10 µm.

Fas joins an impressive list of immunomodulatory proteins that HCMV downregulates from the cell surface by post-translational regulation; others include MHC class-I, MICA, MICB, ULBP2, CD155, CD112, TR1, TR2 and TNFR1 (Baillie *et al.*, 2003; Dunn *et al.*, 2003; Nemčovičová *et al.*, 2013; Prod’homme *et al.*, 2010; Smith *et al.*, 2013; Stern-Ginossar *et al.*, 2007; Tomasec *et al.*, 2005). While MHC class-I, CD112 and MICA (C. Fielding, unpublished) are targeted for efficient proteolytic degradation, CD155, TNFR1, TR2 and Fas are maintained at significant levels within infected cells.

HCMV infection induces resistance to Fas-mediated apoptosis, yet the extent to which this can be attributed to cell surface suppression of Fas will ultimately require the identification of the HCMV gene(s) responsible. Despite systematic screening of an expression library encoding the canonical HCMV genes, the function responsible has yet to be mapped (Seirafian, 2013). In this context, multiple HCMV genes can be expected to impact Fas signalling. The UL36 and UL37 gene products efficiently inhibit Fas-mediated apoptosis by inhibiting caspase-8 activation and cytochrome *c* release, respectively (Arnoult *et al.*, 2004; Goldmacher *et al.*, 1999; Skaletskaya *et al.*, 2001). Moreover, IE2 is known to upregulate c-FLIP, a protease-deficient procaspase-8 homologue (Chiou *et al.*, 2006), whilst the tegument protein UL45 suppresses Fas-mediated killing in the context of HCMV infection by an uncharacterized mechanism (Patrone *et al.*, 2003). These functions operate at or downstream of the DISC, and are thus likely to impact on both TRAIL and Fas-mediated signalling to similar degrees. In addition, since UL141 downregulation of TR2 had a marked impact on TRAIL-mediated cell death (Smith *et al.*, 2013), it is likely that HCMV downregulation of Fas is also an important component of HCMV immune evasion.

Autoimmune lymphoproliferative syndrome (ALPS) is a rare disorder characterized by abnormal lymphocyte survival resulting from a defect in Fas function. A study of two brothers with ALPS experiencing HCMV disease following neonatal exposure documented the development of disseminated infections that were eventually controlled (Arkwright *et al.*, 2000). That Fas-mediated apoptosis is not critical for the control of HCMV disease is consistent with the virus having evolved effective countermeasures to evade Fas-mediated killing. The immune-evasion functions of HCMV are a realistic target for therapeutic intervention.
